# Therapeutic Advances of Stem Cell-Derived Extracellular Vesicles in Regenerative Medicine

**DOI:** 10.3390/cells9030707

**Published:** 2020-03-13

**Authors:** Lei Yin, Xiaotian Liu, Yinghong Shi, Dickson Kofi Wiredu Ocansey, Yuyan Hu, Xiaoxi Li, Chenxiao Zhang, Wenrong Xu, Hui Qian

**Affiliations:** Zhenjiang Key Laboratory of High Technology Research on Exosomes Foundation and Transformation Application, Jiangsu Key Laboratory of Medical Science and Laboratory Medicine, School of Medicine, Jiangsu University, 301 Xuefu Road, Zhenjiang 212013, Jiangsu, China; yinl1882@163.com (L.Y.); lxt18796026236@163.com (X.L.); shiwuyisunny@163.com (Y.S.); dickson.ocansey@ucc.edu.gh (D.K.W.O.); 13777884048@163.com (Y.H.); xiaoxili2010@163.com (X.L.); z565788379@163.com (C.Z.)

**Keywords:** stem cell, extracellular vesicle, tissue damage, regenerative medicine

## Abstract

Extracellular vesicles (EVs), which are the main paracrine components of stem cells, mimic the regenerative capacity of these cells. Stem cell-derived EVs (SC-EVs) have been used for the treatment of various forms of tissue injury in preclinical trials through maintenance of their stemness, induction of regenerative phenotypes, apoptosis inhibition, and immune regulation. The efficiency of SC-EVs may be enhanced by selecting the appropriate EV-producing cells and cell phenotypes, optimizing cell culture conditions for the production of optimal EVs, and further engineering the EVs produced to transport therapeutic and targeting molecules.

## 1. Introduction

Extracellular vesicles (EVs) are vesicular entities with lipid bilayer membranes. They were initially defined as “platelet dust” in 1967 [1]. Intense research regarding EVs in the past half century has enabled a thorough understanding of the origin and biological function of EVs and has positioned EVs on the front line of treatments for various diseases.

EVs exist in all bodily fluids and are produced by all types of cells. Smaller vesicles, known as “exosomes” (EXs), are released from cells through the multivesicular endosomal pathway. Larger vesicles, known as “microvesicles” (MVs), are formed by cell membrane budding and apoptotic bodies are produced by the blebbing of aging or dying cells [2,3]. Apoptotic bodies have been studied less often; thus, EXs and MVs are mainly discussed in this article. EVs can mediate cellular waste degradation and interact with recipient cells through surface receptor binding, endosomal uptake, membrane fusion, membrane protein translocation, and by shuttling RNAs and proteins through vesicle cell channels [2].

EVs carry components of EV-producing cells. They have been shown to exert similar pathophysiological/regenerative effects on tissue and cellular functions when they are applied to experimental animal models. Stem cells are the most common EV-producing cells. Stem cells can be isolated successfully from bone marrow, fat, umbilical cords, embryos, and other tissues. Stem cells can differentiate into many types of cells and they can substitute for injured tissues and fulfill the repair process through the paracrine mechanism at the injury location. Stem cells have been used successfully in the treatment of hematological malignancies, graft-versus-host disease, acute thrombocytopenia, and autoimmune diseases in several experimental in vivo studies [4,5]. However, large-scale production, storage, immune rejection, gene mutation, and tumorigenesis or tumor promotion in vivo limit its application. Stem cell derived-EVs (SC-EVs), as the main paracrine executor, overcome most limitations of stem cell applications. SC-EVs have allowed major advances in preclinical or clinical studies.

In this review, the potential therapeutic applications of SC-EVs in regenerative medicine are discussed and the underlying molecular mechanisms are explored. Some of the possibilities for improving their secretion and altering their components to improve their efficacy toward diverse indications and diseases are summarized.

## 2. Stem Cell-Derived EVs in the Treatment of Damaged Tissue

Numerous preclinical trials have reported that SC-EVs can carry active molecules, such as proteins, lipids, and nucleic acids, and good therapeutic effects against various diseases regarding different systems, including the nervous system, respiratory system, circulatory system, digestive system, urinary system, and others, have been observed.

### 2.1. Neurological System

Brain trauma is a common event that can cause nerve damage and disability. EXs derived from human adipose mesenchymal stem cells (AdMSC-EXs) can significantly increase the number of neurons, reduce inflammation, improve sensory and cognitive function, and produce better effects than AdMSCs alone in rats that have incurred traumatic brain injury (TBI) [6]. Kim et al. indicated that systemic administration of CD63+CD81+ EVs produced by human bone marrow-derived stem cells (BMSC-EVs) decreased neuroinflammation 12 h after a TBI in a mouse model of TBI induced by a controlled cortical impact device [7]. They also found that BMSC-EV infusion preserved the pattern separation and spatial learning abilities of mice, which were demonstrated respectively by an object-based behavioral test and a water maze test [7].

Stroke is the sudden rupture or occlusion of cerebral blood vessels that interrupts the blood supply. It is the main cause of death and disability in Chinese adults. Preclinical studies have shown that SC-EVs seem to be a promising candidate for stroke treatment. Xin et al. showed that infusion of BMSC-EXs enhanced oligodendrogenesis and neurogenesis, remodeled synapses, reduced the incidence of stroke, and accelerated the recovery of neurological functions in a rat model of stroke induced by transient middle cerebral artery occlusion [8]. Webb et al. tested the effect of SC-EVs on stroke in a translational large animal model. In their study, they utilized human neural stem cell-derived EVs (NSC-EVs) to treat ischemic stroke that was manufactured by permanent middle cerebral artery occlusion in pigs, and they found that NSC-EVs eliminated the symptoms of intracranial hemorrhage, decreased the cerebral lesion volume and brain swelling, and preserved the white matter integrity compared to the control pigs [9]. They also indicated that NSC-EV treatment improved behavior and mobility in this model [9]. In an ongoing clinical trial, MSC-EXs were engineered to overexpress miR-124 for the treatment of ischemic stroke (NCT03384433; The estimated primary completion date is April 17, 2020 and the estimated study completion is December 17, 2021).

Alzheimer’s disease (AD) is a common disease that usually occurs in older people. There is no effective treatment for AD. Aβ protein accumulation in nerve cells may promote neurodegeneration and memory impairment in AD. Studies have shown that AdMSC-EXs transport Aβ protease, which effectively inhibits Aβ protein accumulation in nerve cells in vitro, suggesting a possible treatment application for AD [10]. In addition, Cui et al. indicated that BMSC-EX administration ameliorated animal cognitive function and symptoms in a mouse model of AD [11]. In addition, EXs derived from hypoxic-preconditioning BMSCs displayed enhanced therapeutic effects study [11].

The high incidence of spinal cord injury (SCI), which has occurred in recent years, often results in serious sequelae, such as lower limb paralysis and incontinence, for which effective treatments are currently lacking. Liu et al. showed that BMSC-EXs could target specific sites of SCI and promote functional recovery in a rat model of SCI by inhibiting neuronal apoptosis and increasing neuroinflammatory improvement, angiogenesis, and the inactivation of A1 astrocytes [12]. Additionally, Ruppert et al. found that the administration of human BMSC-EVs displayed reduced inflammatory responses, improved motor function, and enhanced mechanical sensitivity thresholds in a rat model of SCI [13]. Recently, a study demonstrated that EXs from human umbilical cord MSCs (hucMSC-EXs) promoted functional recovery in mice with SCI by reducing inflammation [14].

### 2.2. Cardiovascular System

Cardiovascular diseases seriously threaten human health because they have high incidence and mortality rates. Improvement of treatment methods remains a worldwide objective. The proliferation ability of cardiomyocytes is normally considerably weak, and their ability to achieve complete repair through self-regulation after an injury is low. Preclinical studies have indicated that SC-EVs have great application prospects in the treatment of myocardial infarction (MI). Adamiak et al. showed that intravenous injection of induced pluripotent stem cell (iPSC)-derived EVs (iPSC-EVs) at 48 h after MI in mice resulted in improved left ventricular function, reduced left ventricular mass, preservation of viable myocardia and reduction in apoptosis in the infarct zone [15]. A similar phenomenon was observed by Khan who reported that embryonic stem cell (ESC)-EX treatment of mouse MI promoted cardiac blood flow recovery, alleviated myocardial fibrosis, reduced the infarct size, and significantly recovered cardiac function [16]. SC-EVs encapsulate microRNA (miRNA), which reinforces cardiac repair by facilitating angiogenesis. Wang et al. demonstrated that miR-210 enriched in BMSC-EVs drives endothelial proliferation and migration in vitro, and it improves cardiac angiogenesis and cardiac function after MI in mice by targeting Ephrin-A3 [17]. Zhu et al. showed that BMSC hypoxic pretreatment increased the enrichment of angiogenesis-associated miRNAs in BMSC-EXs, which promoted angiogenesis more effectively and improved the therapeutic effects of MI treatment in mice [18].

Preclinical studies have indicated that BMSC-EXs could alleviate inflammatory responses in cardiovascular diseases. Macrophages and neutrophils are activated and recruited to the injured site after a MI and they release a large number of inflammatory factors that trigger a series of inflammatory reactions. In mouse models of doxorubicin-induced dilated cardiomyopathy, intravenous injection of BMSC-EXs improved cardiac function, inhibited cardiac dilation, alleviated cardiomyocyte apoptosis, and reduced the expression of inflammatory factors in addition to the number of proinflammatory macrophages at the infiltration site and in the blood [19].

### 2.3. Respiratory System

Potter et al. showed human BMSC-EVs could significantly reduce pulmonary vascular permeability that was caused by hemorrhagic shock in mice via regulation of cytoskeletal signaling [20]. In another study, Tang et al. showed that BMSC-MVs could promote the stability of the pulmonary vascular structure and improve inflammation in the lungs by delivering angiopoietin-1 (Ang-1) messenger RNAs (mRNAs) in mice. In their research, Tang et al. showed that unmodified BMSC-MVs, but not Ang-1 mRNA-deficient MVs, were able to maintain the integrity of endotoxin-stimulated microvascular endothelial cells in vitro, and they could reduce lung inflammation in a mouse model of lipopolysaccharide (LPS)-induced acute lung injury [21]. Vascular endothelial growth factor (VEGF) can mediate the therapeutic effects of EVs originating from human umbilical cord blood-derived MSCs (hUCB-MSC-EVs) after neonatal hyperoxia-induced lung injury in rats. Ahn et al. demonstrated that treatment with 20 μg of hUCB-MSC-EVs improved lung injury, protected blood vessels, and maintained normal function of the alveoli in newborn rats, whereas VEGF-deficient MSC-MVs functioned poorly [22].

Khatr et al. reported swine BMSC-EVs could disturb the agglutination reaction of various influenza viruses, restrict influenza virus replication in lung epithelial cells in vitro, and alleviate virus-induced apoptosis [23]. The authors suggested that intratracheal administration of BMSC-EVs reduced viral shedding, inhibited lung influenza virus replication, and reduced the production of proinflammatory cytokines in a pig influenza virus model [23]. Additionally, Chaubey demonstrated that intraperitoneal injection of hucMSC-EXs could improve experimental bronchopulmonary dysplasia in mice [24].

### 2.4. Liver

Preclinical studies have demonstrated that SC-EVs harbor the potential to treat liver diseases through the delivery of various active molecules. Haga et al. demonstrated that BMSC-EVs protected against hepatic ischemia/reperfusion injury (IRI) in a mouse model [25]. Similarly, Tamura et al. discovered murine BMSC-EXs improved chemical-induced hepatocyte injury and promoted hepatocyte regeneration in mice, which was mediated through immunosuppression and immune protection [26]. In addition, Rigo et al. discovered human liver SC-EVs effectively reduced liver injury in an ex vivo normothermic hypoxic rat liver perfusion model [27]. Normothermic machine perfusion is an emerging approach for liver preservation before transplantation and it may induce hypoxic injury. Rigo and colleagues indicated that liver SC-EVs reduced the level of alanine aminotransferase and lactate dehydrogenase in perfusate samples, and they protected histological damage and apoptosis in damaged livers [27].

Liver disease often includes liver fibrosis. Our studies have revealed that hucMSC-EXs can relieve liver fibrosis in mice by inactivating transforming growth factor (TGF)-β/Smad signaling, reducing collagen deposition, and alleviating inflammation [28]. Qu et al. also suggested that BMSC-EXs effectively deliver miRNA-181-5p to damaged hepatocyte sites and they prevented liver fibrosis in a mouse model by activating autophagy [29].

Our study reported that hucMSC-EXs carrying glutathione peroxidase1 protect against liver failure in mice by reducing inflammation and oxidative stress [30]. In a murine model of liver failure that was induced by d-galactosamine/tumor necrosis factor-α (TNF-α), Haga et al. found that BMSC-EV administration reduced liver damage and regulated the inflammatory response [31]. In this study, imaging of fluorescence-labeled BMSC-EVs suggested that EVs preferentially migrate to the livers of mice, and their density increases after chemically induced damage. They further demonstrated that EVs from murine or human BMSCs significantly increased the survival rates (murine BMSC-MVs: 57.1%; human BMSC-MVs: 37.5%; Control: 0%) of mice with liver failure, which were superior to those of BMSCs [31]. They demonstrated that Y-RNA-1 is enriched within BMSC-EVs and Y-RNA-1 mediates the protective effects of BMSC-EVs on hepatocyte apoptosis [31].

### 2.5. Diabetes

In a type 2 diabetes rat model, intravenous infusion of hucMSC-EXs decreased the level of blood glucose and promoted glucose uptake, glycolysis, and glycogen storage [32]. Additionally, hucMSC-EX treatment relieved the destroyed islets, activated the insulin-signaling pathway, and ameliorated insulin resistance in the rats [32]. Similarly, Nie et al. found that hucMSC-EXs could protect islet cells from hypoxia-induced injury in vitro, prolong islet cell survival, and improve its function, thus increasing the efficiency of islet transplantation [33].

Shigemoto-Kuroda showed that MSC-EVs could delay the onset of type 1 diabetes (T1D) in mice through immunoregulation. In their study, they found that intravenous injection of MSC-EVs alleviated insulitis, protected insulin-producing cells in the islets, and increased the levels of insulin in plasma. They further demonstrated that MSC-EVs suppressed the progress of T1D mainly by suppressing the activation of antigen-presenting cells and the development of Th1 and Th17 cells [34]. In an in vitro experiment, Favaro et al. found that human BMSC-MV treatment reduced the expressions of IFN-γ and interleukin (IL)-17 and the number of Th17 cells in islet antigen-glutamic acid decarboxylase-pretreated peripheral blood mononuclear cells from patients with T1D. BMSC-MV treatment also increased FoxP3 (+) regulatory T cells, thus suggesting that BMSC-MVs inhibited islet antigen-induced pro-inflammatory responses [35].

Preclinical studies demonstrated that SC-EVs can relieve diabetic complications. Diabetic nephropathy is a common and severe complication in patients with diabetes. In a rat model of STZ-induced diabetic nephropathy, Jiang et al. showed that intravenous infusion of human urinary stem cell-derived EXs (USC-EXs) decreased the level of urinary albumin, alleviated podocyte apoptosis, and promoted glomerular endothelial cell proliferation [36]. Additionally, BMSC-EXs protected damaged neurons and astrocytes in mice, which reduced diabetes-induced cognitive impairment [37].

### 2.6. Eyes

Zhang et al. indicated that retinal inflammation induced by hyperglycemia in rats was reduced after intravitreal injection of hucMSC-EXs, and human retinal endothelial cells displayed downregulated expressions of IL-1β and IL-18 when they were co-cultured with hucMSC-EXs in vitro [38]. They further found that hucMSC-EXs overexpressing miR-126 exhibited improved effects in the same models by targeting high-mobility group box 1 and inhibiting the activity of NLRP3 inflammatory bodies [38]. In addition, He et al. demonstrated that hucMSC-EXs could ameliorate murine retinal laser injury. In their study, hucMSC-EX treatment improved the histological structures of choroidal neovascularization after laser stimulation by downregulating VEGF-A expression, which resulted in better visual function [39].

Preclinical studies have indicated that administration of MSC-EXs can protect against retinal ischemia. In a mouse model of hyperoxic-induced retinopathy, intravitreal injection of MSC-EXs partially preserved retinal vascular blood flow and improved the symptoms of retinal ischemia without immunosuppression [40]. Corneal stromal cells (CSCs) are involved in the formation of a functional corneal endothelium and they play a remarkable role in moderating the corneal endothelial microenvironment [41]. Shen et al. indicated that AdMSC-EX treatment obviously promoted CSC proliferation in vitro, inhibited its apoptosis, triggered higher expression of collagen and fibronectin, and caused lower expression of matrix metalloproteinases [41]. Samaeekia et al. demonstrated that human corneal MSC-derived EXs significantly increased the corneal wound healing rate (Ex vs. Control; 77.5% vs. 41.6%) in a murine model [42], providing a promising non-cellular therapeutic approach for ocular surface injury.

### 2.7. Kidneys

Preclinical studies have demonstrated that SC-EVs have a favorable effect on kidney disease. Yuan et al. revealed that EVs from human iPSC-derived MSCs (hiPSC-MSC-EVs) transported specificity protein (SP1) to renal tubular epithelial cells, increased the expression of sphingosine kinase 1, and inhibited necroptosis, thus preventing renal IRI in rats [43]. In another murine model of IRI, Ranghino et al. found that EVs derived from MSCs within the glomeruli (Gl-MSC-EVs) reduced ischemic damage and facilitated the recovery of murine kidney function post-IRI by promoting tubular epithelial cell proliferation [44].

Additionally, SC-EVs have the potential to ameliorate cisplatin-induced nephrotoxicity. A previous study indicated that hucMSC-EXs transported 14-3-3ζ to renal tubular epithelial cells in vitro and increased ATG16L expression [45]. The interaction of 14-3-3ζ and ATG16L promoted the localization of ATG1L in autophagosome precursors and activated autophagy to prevent acute kidney injury induced by cisplatin in rats [45]. In addition, Tomasoni et al. revealed that BMSC-EXs transported mRNAs of the insulin-like growth factor 1 (IGF-1) receptor to renal tubular epithelial cells in vitro; these mRNAs were then translated into IGF-1 receptor proteins, which could be utilized to increase IGF-1 receptor sensitivity to local IGF-1 and treat cisplatin-induced kidney tubule injury [46].

Eirin et al. used a single intrarenal injection of autologous AdMSC-EVs to treat renal artery stenosis and metabolic syndrome in pigs. In their study, AdMSC-EVs peaked in the stenotic kidney and were taken up by renal tubular epithelial cells on the third day after injection [47]. AdMSC-EV treatment improved renal blood flow, reduced the glomerular filtration rate, alleviated kidney inflammation, and inhibited medullary oxygenation and fibrosis [47]. Eirin et al. discovered that interleukin (IL)-10 in AdMSC-EVs mediates renal protection, as the effect was attenuated after treatment with IL-10–depleted AdMSC-EVs [47]. In addition, BMSC-EVs delivering miR-let7c reduced renal fibrosis in mice by inhibiting renal inflammation [48].

### 2.8. Skin

Skin, as the first line of defense by the body, is in direct contact with the external environment and is easily damaged. Burns are a common type of trauma, which are currently mainly treated with supportive therapy, wound cleaning, and surgical skin grafting. Recent studies have revealed that SC-EVs have great potential to enhance wound healing by delivering anti-inflammatory, antifibrotic, and proangiogenic factors. Kim et al. noted that hUCB-MSC-EXs could be absorbed by mouse skin and promote the synthesis of collagen I and elastin of human keratinocytes and human dermal fibroblasts, which is essential for skin regeneration and is expected to be used in future cosmetics [49]. Our team discovered that hucMSC-EXs transported the Wnt4 protein to activate Wnt/β-catenin signaling and facilitate angiogenesis in a rat model of deep second-degree burn injury, thus shortening wound healing times [50]. We further found that hucMSCs pretreated with a small-molecule drug, 3,3′-diindolylmethane, displayed increasing exosomal Wnt11 autocrine signaling, cell activity, and differentiation potential, eventually leading to a better repair effect in the same model [51].

In a mouse model of a skin defect, Fang et al. found that hucMSC-EXs contain miR-21, miR-23a, miR-125b, and miR-145, which could synthetically antagonize the TGF-β/Smad2 signaling pathway and inhibit the differentiation and accumulation of myofibroblasts, thus reducing scar formation and promoting wound healing [52]. In another study, Chen et al. demonstrated that USC-EXs showed a favorable therapeutic effect on diabetic skin ulcers through that was deleted in malignant brain tumors 1 (DMBT1) [53].

SC-EVs have the potential to alleviate skin aging. Oh et al. showed that human dermal fibroblasts displayed an aging phenotype and increased the synthesis of bovine lactose and matrix degrading enzymes (MMP-1/3) in vitro after ultraviolet irradiation, which was reversed after treatment with hiPSC-EVs [54]. Oh et al. further demonstrated that hiPSC-EVs could reduce the secretion of collagen I of dermal cells and promote cell proliferation and migration, which is essential for improving aging [54].

Additionally, Cho et al. discovered that human AdMSC-EXs could improve atopic dermatitis from house dust mite antigens in a mouse model. In their study, AdMSC-EX treatment reduced the number of eosinophils in the blood and inhibited the infiltration of mast cells and CD86+ and CD206+ cells in skin lesions. The mRNA expression of various inflammatory cytokines, such as IL-4, IL-23, IL-31, and tumor necrosis factor-α (TNF-α), were also reduced in the injured skin [55].

### 2.9. Musculoskeletal System

Bone and cartilage damage is very common clinically, and SC-EVs have exhibited a good therapeutic effect for the repair of bone and cartilage tissue in preclinical experiments. Qi et al. noted that hiPSC-MSC-EXs promoted the expression of osteogenic-related genes in BMSCs and increased cell activity and bone matrix secretion in vitro [56]. They further demonstrated that hiPSC-MSC-EXs significantly stimulated bone regeneration and angiogenesis in a dose-dependent manner in a rat model of critical-sized calvarial defects [56]. In another study, hiPSC-MSC-EXs prevented osteonecrosis of the femoral head of rats through the promotion of angiogenesis [57]. Zhang et al. discovered that hucMSC-EX transplantation dramatically affected bone healing in a rat model of femoral fracture with a similar mechanism [58].

It is difficult for cartilage to self-heal. EV-mediated communications between the BMSCs and juvenile chondrocytes play a crucial role in BMSC repair of cartilage, and the inhibition of EV transport hinders the therapeutic effects of BMSCs [59]. BMSC-EV treated chondrocytes that isolated from patients with osteoarthritis and these cells were stimulated to produce proteoglycan and collagen II [60]. Otsuru et al. demonstrated that MSC-EVs promoted chondrocyte proliferation in vitro and facilitated bone growth in a mouse model of osteogenesis imperfecta by delivering miRNAs, whereas miRNA-deficient MSC-EVs functioned poorly [61]. Zhang et al. reported that EXs produced from human ESC-derived MSCs (ESC-MSC-EXs) delivered CD73 to promote the proliferation and infiltration of chondrocytes in vitro through the activation of AKT and ERK signaling [62]. Preclinical studies showed that SC-EVs could improve inflammation and regulate cartilage homeostasis. Wang et al. noted that ESC-MSC-EXs moderated murine osteoarthritis by equilibrating the synthesis and degradation of cartilage extracellular matrices [63]. Wnt5a is closely involved in the progression of osteoarthritis and is a promising therapeutic target. Mao et al. engineered MSC-EXs to carry miR-92a-3p to reduce the expression of Wnt5a and enhance cartilage formation in mice [64].

### 2.10. Other Diseases

SC-EVs have been considered to be a promising tool for the treatment of reproductive diseases. Zhu et al. demonstrated that AdMSC-EXs encapsulate some proangiogenic miRNAs (miR-126, miR-130a and miR-132) and antifibrotic miRNAs (miR-let7b and miR-let7c) that could induce angiogenesis or decrease fibrosis of the corpus cavernosum in diabetic rats, respectively, thus restoring erectile function [65]. Huang et al. showed that AdMSC-EXs improved ovary function in a mouse model of premature ovarian insufficiency [66]. In their study, AdMSC-EX treatment promoted the activity of human ovarian granule cells in vitro and induced the expression of human ovarian granule cell-associated markers [66]. They also showed that AdMSC-EXs enhanced murine follicle numbers, elevated hormone levels, and improved murine ovarian function by targeting Smad [66]. AdMSC-EXs can exert a protective effect against obesity in mice. Zhao et al. found that AdMSC-EXs delivered active STAT3 to macrophages, which induced macrophage polarization toward an M2 phenotype in vitro [67]. They utilized a mouse model of diet-induced obesity and further demonstrated that AdMSC-EX administration reduced murine inflammation, improved insulin sensitivity, and inhibited hepatic steatosis, which resulted in reduced obesity [67].

**Figure 1:**
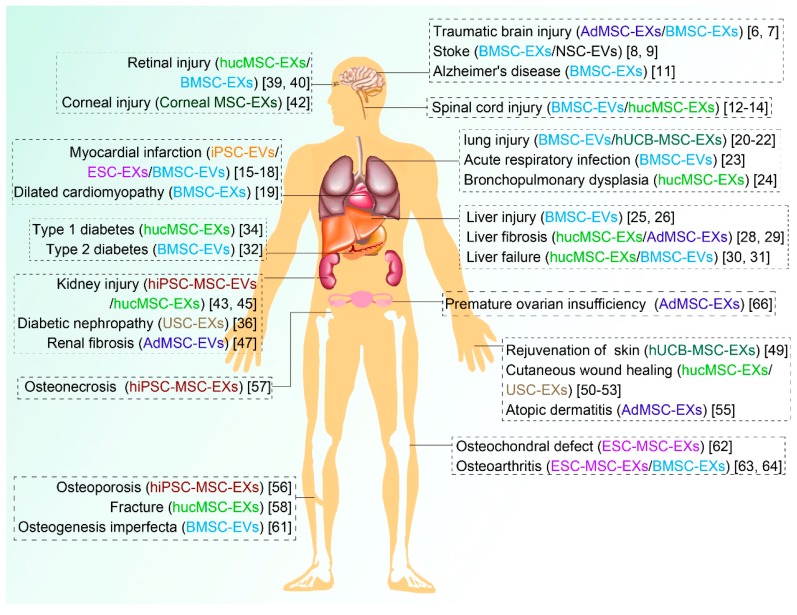
Diseases treated with different types of stem cell-derived EVs. MSC, mesenchymal stem cell; EVs, extracellular vesicles; EXs, exosomes; MVs, microvesicles; AdMSC, adipose-derived MSC; BMSC, bone marrow-derived stem cells; hucMSC, human umbilical cord MSC; ESC, embryonic stem cell; hUCB-MSC, human umbilical cord blood-derived MSC; hiPSC-MSC, human induced pluripotent stem cell-derived MSC; USC, urine-derived stem cell; ESC-MSC, ESC-derived MSC. iPSC, induced pluripotent stem cell.

In conclusion, various preclinical studies have shown that SC-EVs have great potential to stimulate the repair and regeneration of various tissues such as the brain, nerves, heart, lungs, liver, eyes, kidneys, skin, bone, and cartilage (Figure 1) alone and also by carrying active molecules (Table 1). With the continuance of functional research and the expansion of clinical trials, SC-EVs have been shown to have broad application prospects.

## 3. Mechanisms

Some preclinical studies have confirmed that SC-EVs have promise in the field of regenerative medicine. However, the underlying molecular mechanism remains unclear. Exploration of SC-EV involvement in injury repair can contribute to adjustment of SC-EV utilization strategies and accelerate the clinical transformation of SC-EVs. These preclinical investigations have reported that SC-EVs could contribute to the repair of tissue damage through stemness maintenance, induction of regenerative phenotypes, apoptosis inhibition, and immune regulation.

### 3.1. Stemness Maintenance

EVs, which are crucial paracrine components, may induce phenotypic changes in recipient cells and establish functional links between stem cells and recipient tissues under various physiological or pathological conditions. Proliferation, self-renewal, and differentiation of endogenous stem cells are essential for tissue regeneration. SC-EVs encapsulate stem-associated transcription factor mRNAs, including Nanog, Oct4, HoxB4, and Rex-1, which play crucial roles in maintaining stem cell characteristics [68]. In addition, SC-EVs delivering stem cell specific effector molecules containing Wnt3 [68], Hedgehog [69], and others, regulate proliferation, self-renewal, and differentiation of endogenous stem cells.

### 3.2. Induction of Regenerative Phenotypes

Cell proliferation, neovascularization, and nerve regeneration are key phenomena in the healing of damaged tissues. Preclinical studies have demonstrated that SC-EVs can induce regenerative phenotypes to participate in the repair of damaged tissues.

#### 3.2.1. Cell Proliferation

Khan et al. showed that ESC-EXs could protect cardiac progenitor cells and promote cell proliferation in a murine model of MI by delivering miR-294, which eventually led to improved myocardial function [16]. Ranghino et al. indicated that Gl-MSC-EVs prevented IRI in mice by stimulating tubular epithelial cell proliferation [44]. SC-EVs can enhance cell proliferation by carrying proteins to target cells. McBride et al. showed that Wnt3a transported by BMSC-EVs binds to LRP6 receptors to promote dermal fibroblast proliferation in vitro [70]. Similarly, ESC-MSC-EXs can deliver CD73 proteins to activate AKT and ERK signals and improve chondrocyte vitality in vitro [62].

#### 3.2.2. Neovascularization

Mathiyalagan showed that EXs derived from human CD34+ MSCs that transport miR-126-3p to vascular endothelial cells regulated the expression of VEGF, angiopoietin 1/2, matrix metalloproteinases, and thrombospondin 1 in vitro. Additionally, they indicated that the MSC-EXs increased vascular density and improved hind limb motor function in a murine hind limb ischemia-reperfusion model [71]. In another study, Chen et al. indicated that urine-derived SC-EXs carried the proangiogenic protein DMBT1, which promoted angiogenesis and accelerated wound healing in a diabetic mouse model [53].

#### 3.2.3. Nerve Regeneration

BMSC-EXs have been shown to exert improved cognitive and sensorimotor function by promoting endogenous neurogenesis in a rat model of TBI [72]. Mead et al. found that intravitreal injection of BMSC-EXs promoted visual function recovery by facilitating retinal ganglion cell survival and the regeneration of axons via miRNA transfer in a rat optic nerve crush model [73].

### 3.3. Apoptosis Inhibition

SC-EVs can protect against cell apoptosis that is induced by several factors and can reduce tissue damage. Yao et al. revealed that hucMSC-EVs transported mitochondria-located antioxidant enzymes and manganese superoxide dismutase, which inhibited hepatocyte apoptosis induced by oxidative stress and protected against hepatic IRI in rats [74]. EVs produced from amniotic fluid stem cells could capture excess VEGF through VEGFR1 on their surface to protect against VEGF-induced glomerular endothelial cell apoptosis in mice [75].

### 3.4. Immune Regulation

Preclinical studies have confirmed that SC-EVs can wield immunomodulatory effects in the treatment of various diseases by transporting noncoding RNAs, cytokines, and other immunomodulatory molecules. Fujii et al. showed that human BMSC-EVs reduced the ratio of CD62L-CD44+/CS62+CD44-T cells in graft-versus-host diseased mice and prolonged their survival by transporting miR-125a-3p [76]. Li et al. showed that hucMSC-EXs reduced inflammation in burned rats by inhibiting the expression of Toll-like receptor 4 in macrophages [77]. In another study, hucMSCs pretreated with IL-1β produced the EVs that selectively encapsulated miR-146a to induce macrophage polarization toward an M2 phenotype, thus promoting survival in cecal ligation and puncture-induced septic mice [78]. Fetal liver MSC-EXs transport latency-associated peptides, TGF-β, and thrombospondin 1 to activate TGF-β/Smad2/3 signaling in natural killer (NK) cells and they also inhibit NK cell cytotoxicity in vitro, which implies a potential for the treatment of autoimmune diseases [79]. In a rat model of hepatic IRI, hucMSC-EV treatment protected against liver injury by reducing neutrophil inflammatory responses and oxidative stress [74].

**Figure 2:**
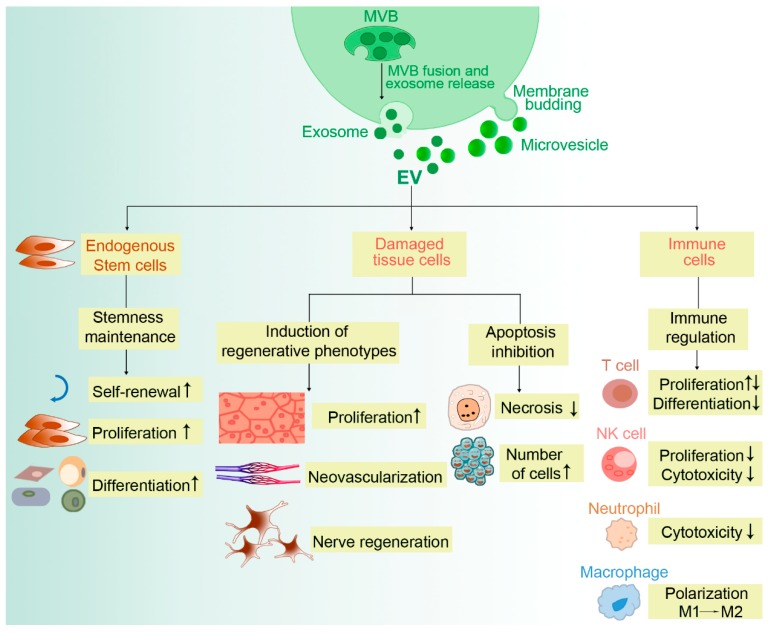
Molecular mechanisms of stem cell-derived EVs in the treatment of disease. Extracellular vesicles (EVs) mainly include exosomes and microvesicles. Exosomes originate from multivesicular bodies (MVB) and microvesicles are formed through cell membrane budding. Stem cell-derived EVs can repair damaged organs and tissues by maintaining stem cell phenotypes, promoting recipient cell proliferation, inhibiting apoptosis, and promoting angiogenesis and nerve regeneration.

In conclusion, SC-EVs are able to repair damaged organs and tissues by promoting recipient cell proliferation, inhibiting apoptosis, facilitating angiogenesis and nerve regeneration, and maintaining stem cell phenotypes (Figure 2). EVs engineered to deliver exogenous biological function molecules and targeting molecules can improve therapeutic efficiency through synergistic effects.

## 4. Stem Cell Culture for Extracellular Vesicle Production

The contents and functions of EVs produced by diverse stem cells vary widely. Some EVs secreted by the same type of stem cells under various culture conditions differ in content and function. The selection of appropriate parental stem cells that are cultured under a specific condition for large-scale production of safer and more effective EVs is essential for the development of new treatments for diseases in the future.

### 4.1. Stem Cell Selection

Stem cells can be successfully isolated from bone marrow, fat, umbilical cords, embryos, placentas, amniotic fluid, blood, livers, skin, and other tissues. The first four types of stem cells listed here based on their origins are currently the most widely studied.

BMSCs exert many biological functions, and their earliest application was for leukemia treatment [80]. Preclinical studies have confirmed that BMSC-EVs can be used to treat various diseases, as expressed in this review. However, BMSC acquirement is difficult and invasive, and BMSCs have poor ability for in vitro expansion, which is not conducive to large-scale production of EVs for clinical use. AdMSCs are easy to obtain and proliferate, and autologous AdMSC transplantation can prevent immune rejection and provide beneficial effects. However, AdMSCs carry the risk of tumor promotion [81]. Therefore, clarifying the effects of AdMSC-EVs on tumors before attempts at clinical application is essential. ESCs are difficult to obtain and utilize for ethical reasons, and they contain the risk of tumorigenesis in the body, which also limits their clinical applications. hucMSCs are easy to acquire and are not associated with ethical controversies. They have strong expansion ability and low immunogenicity. Preclinical studies have reported that hucMSC-EVs have good therapeutic effects in tissue repair and regeneration, as expressed in this review, and hucMSCs have an inhibitory effect on some tumors [81,82]. Currently, commercial cell banks that collect and store hucMSCs are emerging. These advantages enable large-scale production of SC-EVs for clinical applications and iPSC culture makes large-scale production of stem cells easier. Numerous preclinical investigations have demonstrated that iPSC-EVs have similar therapeutic effects to iPSCs in repairing damaged tissue. Therefore, iPSC culture is an alternative for the large-scale production of EVs.

### 4.2. Cell Culture

**Figure 3:**
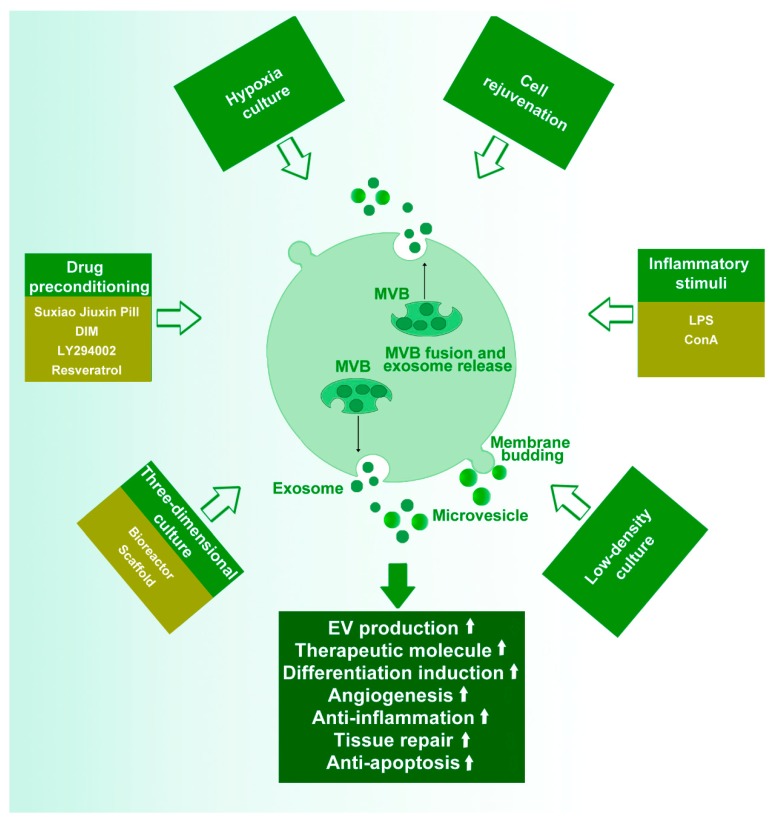
The effect of various culture conditions or treatment on the stem cell-derived EVs. Cell density, cell phenotype, hypoxia treatment, inflammatory stimuli, drug preconditioning, and three-dimensional (3D) culture influence the production and capabilities of extracellular vesicles (EVs). LPS, lipopolysaccharide; DIM, 3,3′-Diindolylmethane; MVB, multivesicular bodies.

Different culture conditions result in different functions and phenotypes of stem cells, which then affect the content and function of EVs. Studies have suggested that various conditions, including cell density, cell phenotype, hypoxia, drug preconditioning, inflammatory stimuli, and 3D culture, influence the properties and activities of stem cells (Figure 3).

#### 4.2.1. Cell Density

Seeding density may affect EV secretion. EVs are typically collected from culture media at 60%–90% cell confluence. Stem cells cultured at high density demonstrate contact inhibition and are induced to enter a resting state [83]. Low-density inoculation (1.5 cells/cm^2^) may result in faster stem cell proliferation [84]. Low-density cultures may activate EV-mediated paracrine signaling of stem cells and promote EV secretion [85].

#### 4.2.2. Cell Phenotypes

Cell phenotypes affect the components and functions of EVs. Stem cells alter phenotypes and functions as the in vitro expansion time increases. Early MSCs have the potential for osteogenic and adipogenic differentiation, and the differentiation potential tilts toward adipogenesis as the in vitro culture is extended [85]. MSCs secrete less EVs after aging [85]. Kulkarni et al. revealed that MVs produced from young BMSCs are rich in autophagy-related gene mRNAs, which better support hematopoietic stem cell transplantation in mice compared to MVs from aging BMSCs [86]. Our unpublished research has indicated that resveratrol treatment improves the senescence phenotype of hucMSCs and increases the differentiation potentials. EXs originating from hucMSCs pretreated with resveratrol had better healing effects on murine lung cancer metastasis than EXs from untreated hucMSCs.

Therefore, improving culture conditions and ensuring stem cells remain as young as possible are necessary, and EVs secreted by younger cells may have superior therapeutic effects on certain diseases. In a different study, Wang et al. noted that EXs produced from MSCs in the late stages of osteogenic differentiation, but not in the early stages, delivered osteogenic differentiation-associated miRNAs to simulate homotypic cell osteogenic differentiation and mineralization in vitro [87]. This provided the hypothesis that stem cells can be induced into a specific tissue phenotype in vitro for collection of their EVs that can be used in corresponding tissue damage repair.

#### 4.2.3. Hypoxia Culture

Cui et al. revealed that EXs produced from hypoxia-preconditioned mouse BMSCs exhibited considerably improved learning and memory capabilities and reduced plaque deposition, neuroinflammation, and Aβ expression in an AD mouse model [11]. Zhu et al. showed that hypoxia (0.5% O_2_) stimulated exosomal mRNA-210 secretion through nSMase2 in mouse BMSCs to promote angiogenesis and improve cardiac function in a mouse model of MI [18].

#### 4.2.4. Drug Preconditioning and Inflammatory Stimuli

Ruan et al. revealed that Suxiao Jiuxin Pill, a type of traditional Chinese medicine, could promote mouse BMSC-EV secretion [88]. Our study revealed that 3,3′-Diindolylmethane-stimulated hucMSCs secreted Wnt11-overexpressing EVs that could be used to improve wound healing in rats [51]. In addition, Kulkarni et al. noted that aging BMSCs treated with AKT signal inhibitor LY294002 reduced exosomal miR-17 and miR-34a, and both downregulated autophagy-associated mRNA expression in recipient cells [86]. LY294002 treatment increased autophagy-associated genes in BMSC-MVs [86]. MVs from aging BMSCs that were pretreated with LY294002 were more powerful than MVs from young BMSCs in supporting hematopoietic stem transplantation in mice [86]. In another study, EVs from human BMSCs activated by LPS or ConA displayed enhanced anti-inflammatory ability and reduced the release of TNF-α and IFN-γ from spleen cells in vitro [89].

#### 4.2.5. Three-Dimensional Culture

Cell culture configurations for producing EVs include both 2D and 3D systems. The 2D system uses conventional polystyrene flasks for the adhesion growth of EV-producing cells and the 3D system for EV production mainly includes a scaffold-free bioreactor and scaffold-based approach [90]. The 3D system can bestow EV production with higher yields, more natural features, and better therapeutic effects compared to the 2D system [90].

Bioreactors are typically used for large-scale production of EVs. In these devices, EV-producing cells were sown into cylindrical hollow fibers that provided a high surface area that was available to billions of cells. These cells were seeded into the bioreactor and could produce 4-fold more EVs than cells cultured in a traditional 2D flask [91]. EXs from stem cells cultured in a bioreactor protected human dopaminergic neurons from apoptosis in vitro, which was not observed in EXs collected from the 2D culture [92]. In a rat model of experimental TBI, EXs from BMSCs cultured in 3D scaffolds exhibited greater spatial learning ability than EVs harvested from 2D conditions [93].

## 5. Bioactive Molecule Delivery

EVs are nanolevel active ingredients that are secreted by cells. These cells actively release the vesicles into the internal environment to participate in intercellular substance transport and signal transduction. Preclinical trials have shown that EVs are highly biocompatible and safe and can cross the blood–brain barrier [8]. EVs engineered to load bioactive molecules can prolong the plasma circulation half-life of active ingredients. These advantages make EV an ideal drug delivery tool.

### 5.1. Biodistribution of EVs

The biodistribution of EVs affects their efficiency in the delivery of therapeutic entities as part of the treatment of various diseases. Abello et al. analyzed the biodistribution of EXs by labeling hucMSC-EXs with gadolinium lipid (GdL-EXs) or a near-infrared dye; i.e., 1,1′-dioctadecyl-3,3,3′,3′- tetramethylindotricarbocyanine iodide (DiR-EXs), in tumor bearing mice [94]. In this study, they found that GdL-EXs mainly accumulated in the liver (38%), tumor (18%) and kidney (8%) at 24 h after intravenous injection, and DiR-EXs mostly appeared in the liver, spleen, and tumor at 48 h after intravenous administration [94]. DiR-EXs displayed longer circulation times than PEGylated nanoparticles [94]. In another study, Wiklander et al. observed a similar distribution where DiR-labeled EVs mainly appeared in the liver and spleen in mouse tissue at 24 h after intravenous injection [95]. They further indicated that EV dose and the approach of the injection influenced the EV biodistribution [95]. Intravenous infusion of lower EV doses demonstrated relatively higher liver accumulation in comparison with higher doses [95]. Compared to intravenous infusion of EVs, subcutaneous and intraperitoneal injection facilitated EV accumulation in the pancreas and gastrointestinal tract, but inhibited EV distribution in the liver and spleen [95]. This suggested that the flexible choice of EV injection doses and injection methods will benefit therapeutic studies.

### 5.2. Delivery of Therapeutic Molecules

**Figure 4:**
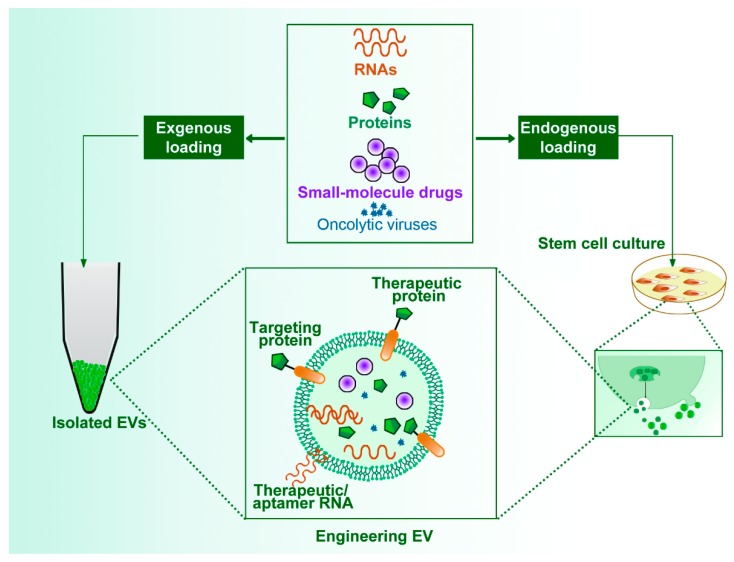
Engineering EVs. Extracellular vesicles (EVs) can deliver therapeutic entities, including proteins, RNAs, oncolytic viruses and small-molecule drugs, with endogenous loading during EV biogenesis or exogenous loading after EV isolation. The engineered EVs can express targeting peptides or therapeutic proteins on their surfaces and bind aptamers or therapeutic RNAs through RNA-binding proteins. Additionally, EVs can encapsulate these therapeutic entities and protect them from degradation or failure.

Various preclinical studies have reported that EVs can transport nucleic acids, proteins, oncolytic viruses, and small-molecule drugs to treat and target various diseases. These substances have great application prospects (Figure 4).

#### 5.2.1. Nucleic Acids

mRNAs, miRNAs, small interfering RNAs (siRNAs), and circular RNAs can be transported in EVs. In 2007, Valadi et al. first noted that EXs were involved in the exchange of mRNAs and miRNAs between cells [96]. Even when recipient cells are derived from other species, mRNAs and miRNAs can function in their new positions through EX transport [96]. This suggests that EVs have the potential to participate in a drug delivery system.

Rat BMSC-EXs loaded with miRNA-133b can be used for neurological recovery after stroke in rats [97]. In another study, iPSC-EXs delivering siRNA inhibited intracellular adhesion molecule-1 expression and reduced neutrophil adhesion in lung microvascular endothelial cells in vitro [98].

Kojima et al. employed several devices that enable efficient and customizable production of designer EXs in engineered cells [99]. These devices in EX-originated cells enhanced EX production through combined expression of STEAP3, syndecan-4, and a fragment of L-aspartate oxidase, they promoted specific mRNA packaging through L7Ae and CD63 coexpression and increased the delivery rate of mRNA into target cells through the expression of constitutively active mutant S368A of connection [99]. Kojima et al. combined these devices with a targeting module; i.e., RVG-Lamp2b, to treat a mouse model of Parkinson’s disease, resulting in attenuated neurotoxicity and neuroinflammation [99].

#### 5.2.2. Proteins

It was documented earlier that EXs derived from 14-3-3ζ-overexpressing hucMSCs could deliver 14-3-3ζ proteins to protect against cisplatin-induced nephrotoxicity in rats [45]. In addition, hucMSC-EXs were used for transporting Wnt11 proteins to promote wound healing in rats [51].

EVs could be modified to overexpress CD47, which provided a “do not eat me” signal, and the activation of this signal causes EXs to escape phagocytosis [100], thus increasing the residence time of EVs in the body. In other research, EXs carrying TAT peptides were used to target nuclei and EXs mediating Arg-Gly-Asp (RDG) peptides operating to induce cancer cell apoptosis in mice [101].

#### 5.2.3. Small-Molecule Drugs

Sun et al. found that EVs had good drug-loading capability. They showed that EVs and curcumin mixtures were more effective than curcumin in improving inflammation in a mouse model of LPS-induced septic shock [102]. An underlying limiting factor in EV drug delivery is the drug-loading efficiency. Stem cells are more tolerant of chemotherapeutic drugs than other cells due to EV-mediated drug effluxes. Using this mechanism, MSCs were exposed to high concentrations of paclitaxel and their EVs were harvested, which encapsulated considerable amounts of paclitaxel [103]. In another study, hucMSC-EVs loaded with vincristine provided more cytotoxicity than the free drug [104]. Perteghella et al. used silk/curcumin to create nanoparticles with an average diameter of 100 nm and incubated MSCs with those nanoparticles [105]. MSCs could then produce and release EVs that encapsulated the silk/curcumin nanoparticles. This strategy can be used for the delivery of curcumin [105]. In addition, Tian et al. used engineered EXs that expressed alpha integrin-specific iRGD to load doxorubicin via electroporation and they demonstrated that doxorubicin was specifically delivered to tumor tissues, which resulted in the inhibition of tumor growth in mice [106].

#### 5.2.4. Oncolytic Viruses

EVs can be utilized as vectors to deliver oncolytic viruses to tumor sites [107]. Oncolytic viruses are promising in the treatment of various cancers because they selectively infect cancer cells, synchronously stimulate the immune response and attract more immune cells to continue to kill residual cancer cells [108,109,110]. However, delivery of oncolytic viruses to tumor sites remains a major challenge. Garofalo et al. demonstrated that oncolytic viruses could be delivered by tumor-derived EVs (T-EVs), which increases their antitumor properties and displays a selective tropism for any tumor sites, independent of the tumor type originating the EVs [110]. They further found that tumor tropism is achieved when oncolytic viruses are loaded inside T-EVs that are injected intravenously, but not when administrated intraperitoneally [111]. In addition, they indicated that T-EVs delivering oncolytic viruses did not harm other body tissues and displayed enhanced anti-tumor ability after the process of combined loading with paclitaxel in mice [112]. However, T-EVs are not as desirable as SC-EVs in the treatment of various diseases due to their production limitation and tumor-promoting properties. We speculate that SC-EVs can also deliver oncolytic viruses to fight against tumors. It has been reported that stem cells display tropism for the tumor sites and could transfer oncolytic viruses to tumor cells specifically [113,114]. Sonabend et al. found that MSCs could deliver a conditionally replicating oncolytic adenovirus to murine gliomas [113]. In another study, Hoyos et al. used MSCs to transfer oncolytic and apoptotic adenoviruses in a non-small-cell lung cancer mouse model [114]. They found that MSCs traveled to lung tumors replicated and released both viruses to the tumor microenvironment, displayed enhanced antitumor activity, and prolonged survival of tumor-bearing mice [114]. SC-EVs have more advantages than stem cells and they also display tropism for tumor sites [94]. Tests of their virus-delivery ability is promising in the field of oncotherapy.

## 6. Conclusions and Prospects

SC-EV treatment has made great progress in the field of regenerative medicine, and a large number of preclinical experiments have laid a solid foundation for its clinical transformation application. Selecting suitable EV-producing cells, optimizing cell culture conditions and separation techniques, and using EVs to transport biomolecules or small-molecule drugs can improve their efficacy toward diverse indications and diseases.

However, many hurdles remain for clinical applications of EVs. Choosing a type of appropriate EV-producing cells is of utmost importance. Current research mainly focuses on the treatment of a limited number of diseases in the field of regenerative medicine and oncology by using SC-EVs. The functions of SC-EVs must be tested for many other diseases. Furthermore, research on the functions of EVs in other cells such as epithelial, endothelial progenitor, and red blood cells must be accelerated and expanded.

To achieve higher quality EVs, EV-producing conditions must be optimized, including the appropriate selection of culture medium, cell seeding density, cell phenotype, culture time, media-collecting time, separation technology, and other parameters of EV production for further research into the various therapeutic purposes of EVs.

The drug-loading potential of SC-EVs must be further explored. Currently, genome-editing techniques facilitate EV engineering with diverse content and functions but may introduce uncertain mutations in EV-producing cells, which in turn affects the content and functions of corresponding EVs. Therefore, improving the safety and operability of genome-editing techniques, reducing off-target efficiency, and ultimately accurately producing EVs with specific functions and specific components are necessary.

Improving the drug-loading efficiency of EV through another method is crucial. Exogenous drugs are currently loaded into EVs mainly through electroporation but the efficiency of this technique remains unsatisfactory. Although drug-loading efficiency is not necessarily proportional to therapeutic efficiency, balancing these two types of efficiency is advisable. Therefore, the optimal concentrations of drugs with the lowest side effects to achieve the highest therapeutic efficiency may be preferable. For this, we still must improve drug-loading efficiency.

Quantitative techniques for EV efficacy remain to be developed and improved. As a heterogeneous group, EVs are susceptible to external factors such as culture, isolation, and storage. Therefore, methods for quantifying the efficacy of EVs must be considered in the future.

The advantages of SC-EVs and their excellent application potential are driving the advancement of regenerative medicine. The future development goal is to optimize EV-producing conditions, improve production technology, improve yield and quality, quantify their therapeutic efficacy, engineer operations to endow EVs with more therapeutic functions, and promote their clinical transformation to enable them to ultimately benefit humans in a wider field.

## Figures and Tables

**Table 1:** Experimental model diseases treated with different types of stem cell-derived EVs. Cell proliferation, neovascularization, and nerve regeneration are key phenomena in healing of damaged tissues. Stem cell-derived extracellular vesicles (SC-EVs) can participate in the repair of damage through stemness maintenance, induction of regenerative phenotypes, apoptosis inhibition, and immune regulation. MSC, mesenchymal stem cell; EXs, exosomes; MVs, microvesicles; AdMSC, adipose-derived MSC; BMSC, bone marrow-derived stem cells; hucMSC, human umbilical cord MSC; ESC, embryonic stem cell; EnMSC, endometrium-derived MSC; hUCB-MSC, human umbilical cord blood-derived MSC; hiPSC-MSC, human induced pluripotent stem cell-derived MSC; ESC-MSC, ESC-derived MSC; NSC, neural stem cell; i.v., intravenous injections; s.c., subcutaneous injection; i.p., intraperitoneal injection; bw, body weight.

Indication	Species/Tissue	EV Source/Injection Method	Dose	Main Outcome in Target Disease/Injured Tissue	Mechanism	Reference
**Neurological system**						
Traumatic brain injury (TBI)	Rat	Human AdMSC-EXs/intravenous injections (i.v.)	100 μg	Recovery of motor behavior function and cortical brain injury↓	Delivering MALAT1	[6]
TBI	Mouse	Human BMSC-EXs/i.v.	30 µg	Suppression of neuroinflammation, improvement of cognitive function	Unknown	[7]
Stroke	Rat	Rat BMSC-EXs/i.v.	100 μg	Improved functional recovery, neurite remodeling↑	Neurogenesis↑, angiogenesis↑	[8]
Stroke	Pig	NSC-EVs/i.v.	Unknown	Improvement of neural tissue preservation and functional levels	Unknown	[9]
Alzheimer’s disease	Mouse	EXs from hypoxia-stimulated BMSCs/i.v.	150 µg	Learning and memory capabilities↑	Restoration of synaptic dysfunction and regulation of inflammatory responses via miR-21	[11]
Spinal cord injury (SCI)	Rat	BMSC-EXs/i.v.	200 µg	Angiogenesis↑, neuronal cell apoptosis↓, glial scar formation↓, lesion size↓, inflammation↓, axonal regeneration↑, improvement of functional behavioral recovery effects	Activation of A1 neurotoxic reactive astrocytes↓	[12]
SCI	Rat	Human BMSC-EVs/i.v.	1 × 10^9^ particles	Inflammatory response↓, improved motor function, enhanced mechanical sensitivity threshold	Unknown	[13]
SCI	Mouse	hucMSC-EXs/i.v.	20 µg and 200 µg	Improving functional recovery	Inflammation↓	[14]
**Cardiovascular system**						
Myocardial infarction (MI)	Mouse	iPSC-EVs/i.v	Unknown	Improvement of left ventricular function, left ventricular mass↓, preservation of viable myocardium	Delivery of ESC specific miR-294	[15]
MI	Mouse	Mouse ESC-EXs/intramyocardial injection	Two separate 10 μL injections	Neovascularization↑, cardiomyocyte survival↑, fibrosis post infarction↓, resurgence of cardiac proliferative response	Delivering miR-294	[16]
MI	Mouse	Mouse BMSC-EVs/i.v.	Unknown	Improving cardiac function, angiogenesis↑	Delivering miR-210	[17]
MI	Mouse	EXs derived from hypoxia-stimulated BMSC/intramyocardial injection	EXs derived from 2 × 10^7^ MSCs, in 30 μL PBS	Survival↑, scar size↓, better cardiac functions recovery	miR-210 and neutral sphingomyelinase 2 activities↑	[18]
Dilated cardiomyopathy	Mouse	Mouse BMSC-EXs/i.v.	300 μg	Improving cardiac function, attenuating cardiac dilation, cardiomyocytes apoptosis↓, inflammatory cells infiltration↓	Regulating the polarization of the macrophage	[19]
**Respiratory system**						
Lung injury	Mouse	Human BMSC-EVs/i.v.	30 μg	Lung vascular permeability↓	Modulating cytoskeletal signaling	[20]
Acute lung injury	Mouse	Human BMSC-MVs	Unknown	Histological injury↓, pulmonary capillary permeability↓	Delivering Angiopoietin-1 mRNA and immune regulation	[21]
Neonatal hyperoxic lung injury	Rat	hUCB-MSC-EVs/intratracheal injection	20 μg	Impaired alveolarization and angiogenesis↓, cell death↓, activated macrophages↓, proinflammatory cytokines↓	Transfer of VEGF protein	[22]
Acute respiratory infection	Pig	Swine BMSC-EVs/intratracheal injection	80 μg/kg bw	The hemagglutination activity of viruses↓, virus shedding and replication↓, proinflammatory cytokines↓, influenza virus-induced lung lesions↓	Transfer of RNAs	[23]
Bronchopulmonary dysplasia	Mouse	hucMSC-EXs/i.p.	4.5 × 108 and 2.88 × 107 particles	Improvement in pathology, pulmonary inflammation↓, alveolar-capillary leakage↓, chord length↓, alveolar simplification↓	Transfer of TSG-6 protein	[24]
**Liver**						
Hepatic ischemia/reperfusion injury	Mouse	Mouse BMSC-EVs/i.v.	2 × 1010 particles	Tissue necrosis↓, apoptotic cells↓, serum aminotransferase↓, NACHT mRNA↑, LRR mRNA↑, PYD domains-containing protein 12 mRNA↑, chemokine ligand 1 mRNA↑, mRNA expression of inflammatory cytokines↓	Modulation of inflammatory response	[25]
Liver injury	Mouse	Mouse BMSC-EVs/i.v.	10 μg	ALT↓, liver necrotic areas↓, apoptotic cells↓, cell proliferation↑, the mRNA expression of anti-inflammatory cytokines↑, the number of Treg cells↑	Immunosuppression and immune protection	[26]
Liver fibrosis	Mouse	hucMSC-EXs/intrahepatic injection	250 μg	Surface fibrous capsules↓, textures soft↑, inflammation and collagen deposition↓, serum aspartate aminotransferase activity↑, collagen types I and III, TGF-β and phosphorylation Smad2 expression↓	Inhibiting EMT and protecting hepatocytes	[28]
Liver fibrosis	Mouse	miRNA-181-5p-overexpressing AdMSC-EXs/intrasplenic injection	40 μg	Collagen I, vimentin, α-SMA and fibronectin expression↓	Transfer of miRNA-181-5p and autophagy activation	[29]
Liver failure	Mouse	hucMSC-EXs/i.v.	16 mg/kg bw	Rescuing liver failure, oxidative stress and apoptosis↓	Transfer of glutathione peroxidase1	[30]
Liver failure	Mouse	Mouse and human BMSC-EVs/i.v. or i.p.	2 × 108 to 2 × 1010 particles	Hepatic injury↓, modulating cytokine expression, survival↑	Transfer of noncoding RNA Y-RNA-1	[31]
**Diabetes**						
Type 2 diabetes	Ra	hucMSC-EXs/i.v.	10 mg/kg bw	Blood glucose↓; glucose uptake, glycolysis and glycogen storage↑	Reversing insulin resistance and protecting islets	[32]
Type 1 diabetes	Mouse	Human BMSC-derived EVs/i.v.	30 μg	Inactivation of antigen-presenting cells, development of Th1 and Th17 cells↓	Immunoregulation	[34]
**Eyes**						
Retinal laser injury	Mouse	hucMSC-EXs	50, 100, and 150 ng	Improving the histological structures of choroidal neovascularization	Downregulation of VEGF-A.	[39]
Oxygen-induced retinopathy	Mouse	Human BMSC-EXs/intravitreal injection	20 μg	Preserving retinal vascular flow and improving the symptoms of retinal ischemia	Unknown	[40]
Corneal epithelial wound healing	Mouse	Human Corneal Mesenchymal Stromal Cell-derived EXs	5 × 10^6^ particles	Increasing the corneal wound healing rate	Unknown	[42]
**Kidneys**						
Diabetic nephropathy	Rat	Human urinary stem cell-derived EXs/i.v.	65 mg/kg bw	Urinary albumin↓, preventing kidney injury	Inhibition of podocyte apoptosis and promotion of glomerular endothelial cell proliferation	[36]
Renal ischemia/reperfusion injury	Rat	hiPSC-MSC-EVs/i.v.	1 × 1012 particles	Histological score↓, serum levels of creatinineand urea nitrogen↓, oxidative stress↓	Exosomal SP1 activating the expression of SK1 and the generation of S1P	[43]
Cisplatin-induced acute kidney injury	Rat	hucMSC-EVs/intrarenal injection	200 μg/kidney	Histological injury↓, apoptosis↓, proliferation↑, serum levels of creatinine and urea nitrogen↓	Exosomal 14-3-3zeta interacting with ATG16L and autophagy activation	[45]
Renal fibrosis	Pig	Autologous AdMSC-EVs/intrarenal injection	1 × 1010 particles	Renal inflammation↓, medullary oxygenation and fibrosis↓, restoring renal blood flow and glomerular filtration rate	Transfer of IL-10 protein	[47]
**Skin**						
Rejuvenation of skin	Human skin tissues	hUCB-MSC-EXs/coculture	1 × 109 particles/mL	EXs approaching the epidermis, expressions of Collagen I and Elastin↑	Unknown	[49]
Wound healing	Rat	hucMSC-EXs/s.c.	200 μg	Re-epithelialization↑, expression of CK19, PCNA, collagen I (compared to collagen III)↑	Transfer of Wnt4 and Wnt11, and activation of Wnt/β-catenin and AKT pathway	[50,51]
Wound healing	Mouse	hucMSC-EXs/s.c.	100 μg/mL	Scar formation and myofibroblast accumulation↓	Transfer of specific microRNAs (miR-21, -23a, -125b, and -145) and suppression of TGF-β/Smad2 pathway	[52]
Chronic non-healing wounds	Mouse	Human urinary stem cell-derived EXs/s.c.	200μg	Soft tissue wound healing↑	Transfer of DMBT1 protein and angiogenesis	[53]
Atopic dermatitis	Mouse	Human AdMSC-EXs/i.v. or s.c.	0.14 μg, 1.4 μg, and 10 μg	Clinical score↓, serum IgE↓, the number of eosinophils in blood↓, the infiltration of mast cells, CD86+, and CD206+ cells in skin lesions↓, expression of inflammatory cytokines↓	Unknown	[55]
**Musculoskeletal system**						
Osteoporosis	Rat	hiPSC-MSC-EXs/i.v.	1 × 1010 or 1 × 109 particles	Preventing bone loss, microvessel density in the femoral head↑	Activation of the PI3K/Akt signaling pathway	[56]
Osteonecrosis	Rat	hiPSC-MSC-EXs/intracranial implantation	200 μg	Bone regeneration↑	Angiogenesis and osteogenesis↑	[57]
Stabilized fracture	Rat	hucMSC-EXs/injection near the fracture site	100 μg/mL	Angiogenesis and bone healing↑	HIF-1alpha-mediated promotion of angiogenesis	[58]
Osteogenesis imperfecta	Mouse	Murine BMSC-EVs/i.v.	300 uL	Facilitating bone growth	Delivery of miRNAs	[61]
Osteochondral defect	Rat	Human ESC-MSC-EXs/intra-articular injection	100 μg	Cellular proliferation and infiltration↑, matrix synthesis↑, displaying a regenerative immune phenotype	Exosomal CD73 mediating adenosine activation of AKT and ERK signaling	[62]
Osteoarthritis	Mouse	Human ESC-MSC-EXs/intra-articular injection	5 μL	Cartilage destruction and matrix degradation↓	Balance of synthesis and degradation of cartilage extracellular matrix	[63]
Osteoarthritis	Mouse	miR-92a-3p-overexpressing BMSC-EXs	15 μL (500 μg/mL)	Chondrogenesis↑, cartilage degradation↓	Targeting Wnt5A	[64]
**Other diseases**						
Erectile dysfunction	Rat	Rat AdMSC-EXs/orthotopic injection	10 and 100 μg	Promoting angiogenesis and decreasing fibrosis in the corpus cavernosum	Delivery of proangiogenic miRNAs and antifibrotic miRNAs	[65]
Premature ovarian insufficiency	Mouse	Human AdMSC-EXs	An approximate amount produced by 1 × 10^6^ AdMSCs	Enhancing follicle numbers, elevating hormone levels, and improving ovarian function	Inhibition of Smad expression	[66]
Obesity	Mouse	Mouse AdMSC-EXs/i.p.	30 μg	Reducing obesity and inflammation	Improving insulin sensitivity and inhibiting hepatic steatosis	[67]

## References

[1] Hargett L.A., Bauer N.N. (2013). On the origin of microparticles: From “platelet dust” to mediators of intercellular communication. Pulm. Circ..

[2] Wiklander O.P.B., Brennan M.A., Lotval J., Breakefield X.O., El Andaloussi S. (2019). Advances in therapeutic applications of extracellular vesicles. Sci. Transl. Med..

[3] Andaloussi S.E., Mager I., Breakefield X.O., Wood M.J. (2013). Extracellular vesicles: Biology and emerging therapeutic opportunities. Nat. Rev. Drug Discov..

[4] Dias I.E., Pinto P.O., Barros L.C., Viegas C.A., Dias I.R., Carvalho P.P. (2019). Mesenchymal stem cells therapy in companion animals: Useful for immune-mediated diseases?. BMC Vet. Res..

[5] Abdelrazik H., Giordano E., Barbanti Brodano G., Griffoni C., De Falco E., Pelagalli A. (2019). Substantial Overview on Mesenchymal Stem Cell Biological and Physical Properties as an Opportunity in Translational Medicine. Int. J. Mol. Sci..

[6] Patel N.A., Moss L.D., Lee J.Y., Tajiri N., Acosta S., Hudson C., Parag S., Cooper D.R., Borlongan C.V., Bickford P.C. (2018). Long noncoding RNA MALAT1 in exosomes drives regenerative function and modulates inflammation-linked networks following traumatic brain injury. J. Neuroinflamm..

[7] Kim D.K., Nishida H., An S.Y., Shetty A.K., Bartosh T.J., Prockop D.J. (2016). Chromatographically isolated CD63+CD81+ extracellular vesicles from mesenchymal stromal cells rescue cognitive impairments after TBI. Proc. Natl. Acad. Sci. USA.

[8] Xin H., Katakowski M., Wang F., Qian J.Y., Liu X.S., Ali M.M., Buller B., Zhang Z.G., Chopp M. (2017). MicroRNA cluster miR-17-92 Cluster in Exosomes Enhance Neuroplasticity and Functional Recovery After Stroke in Rats. Stroke.

[9] Webb R.L., Kaiser E.E., Jurgielewicz B.J., Spellicy S., Scoville S.L., Thompson T.A., Swetenburg R.L., Hess D.C., West F.D., Stice S.L. (2018). Human Neural Stem Cell Extracellular Vesicles Improve Recovery in a Porcine Model of Ischemic Stroke. Stroke.

[10] De Godoy M.A., Saraiva L.M., de Carvalho L.R.P., Vasconcelos-Dos-Santos A., Beiral H.J.V., Ramos A.B., Silva L.R.P., Leal R.B., Monteiro V.H.S., Braga C.V. (2018). Mesenchymal stem cells and cell-derived extracellular vesicles protect hippocampal neurons from oxidative stress and synapse damage induced by amyloid-beta oligomers. J. Biol. Chem..

[11] Cui G.H., Wu J., Mou F.F., Xie W.H., Wang F.B., Wang Q.L., Fang J., Xu Y.W., Dong Y.R., Liu J.R. (2018). Exosomes derived from hypoxia-preconditioned mesenchymal stromal cells ameliorate cognitive decline by rescuing synaptic dysfunction and regulating inflammatory responses in APP/PS1 mice. FASEB J..

[12] Liu W., Wang Y., Gong F., Rong Y., Luo Y., Tang P., Zhou Z., Xu T., Jiang T., Yang S. (2019). Exosomes Derived from Bone Mesenchymal Stem Cells Repair Traumatic Spinal Cord Injury by Suppressing the Activation of A1 Neurotoxic Reactive Astrocytes. J. Neurotrauma.

[13] Ruppert K.A., Nguyen T.T., Prabhakara K.S., Toledano Furman N.E., Srivastava A.K., Harting M.T., Cox C.S., Olson S.D. (2018). Human Mesenchymal Stromal Cell-Derived Extracellular Vesicles Modify Microglial Response and Improve Clinical Outcomes in Experimental Spinal Cord Injury. Sci. Rep..

[14] Sun G., Li G., Li D., Huang W., Zhang R., Zhang H., Duan Y., Wang B. (2018). hucMSC derived exosomes promote functional recovery in spinal cord injury mice via attenuating inflammation. Mater. Sci. Eng. C Mater. Biol. Appl..

[15] Adamiak M., Cheng G., Bobis-Wozowicz S., Zhao L., Kedracka-Krok S., Samanta A., Karnas E., Xuan Y.T., Skupien-Rabian B., Chen X. (2018). Induced Pluripotent Stem Cell (iPSC)-Derived Extracellular Vesicles Are Safer and More Effective for Cardiac Repair Than iPSCs. Circ. Res..

[16] Khan M., Nickoloff E., Abramova T., Johnson J., Verma S.K., Krishnamurthy P., Mackie A.R., Vaughan E., Garikipati V.N., Benedict C. (2015). Embryonic stem cell-derived exosomes promote endogenous repair mechanisms and enhance cardiac function following myocardial infarction. Circ. Res..

[17] Wang N., Chen C., Yang D., Liao Q., Luo H., Wang X., Zhou F., Yang X., Yang J., Zeng C. (2017). Mesenchymal stem cells-derived extracellular vesicles, via miR-210, improve infarcted cardiac function by promotion of angiogenesis. Biochim. Biophys. Acta Mol. Basis Dis..

[18] Zhu J., Lu K., Zhang N., Zhao Y., Ma Q., Shen J., Lin Y., Xiang P., Tang Y., Hu X. (2018). Myocardial reparative functions of exosomes from mesenchymal stem cells are enhanced by hypoxia treatment of the cells via transferring microRNA-210 in an nSMase2-dependent way. Artif. Cells Nanomed. Biotechnol..

[19] Sun X., Shan A., Wei Z., Xu B. (2018). Intravenous mesenchymal stem cell-derived exosomes ameliorate myocardial inflammation in the dilated cardiomyopathy. Biochem. Biophys. Res. Commun..

[20] Potter D.R., Miyazawa B.Y., Gibb S.L., Deng X., Togaratti P.P., Croze R.H., Srivastava A.K., Trivedi A., Matthay M., Holcomb J.B. (2018). Mesenchymal stem cell-derived extracellular vesicles attenuate pulmonary vascular permeability and lung injury induced by hemorrhagic shock and trauma. J. Trauma Acute Care Surg..

[21] Tang X.D., Shi L., Monsel A., Li X.Y., Zhu H.L., Zhu Y.G., Qu J.M. (2017). Mesenchymal Stem Cell Microvesicles Attenuate Acute Lung Injury in Mice Partly Mediated by Ang-1 mRNA. Stem Cells.

[22] Ahn S.Y., Park W.S., Kim Y.E., Sung D.K., Sung S.I., Ahn J.Y., Chang Y.S. (2018). Vascular endothelial growth factor mediates the therapeutic efficacy of mesenchymal stem cell-derived extracellular vesicles against neonatal hyperoxic lung injury. Exp. Mol. Med..

[23] Khatri M., Richardson L.A., Meulia T. (2018). Mesenchymal stem cell-derived extracellular vesicles attenuate influenza virus-induced acute lung injury in a pig model. Stem Cell. Res. Ther..

[24] Chaubey S., Thueson S., Ponnalagu D., Alam M.A., Gheorghe C.P., Aghai Z., Singh H., Bhandari V. (2018). Early gestational mesenchymal stem cell secretome attenuates experimental bronchopulmonary dysplasia in part via exosome-associated factor TSG-6. Stem Cell. Res. Ther..

[25] Haga H., Yan I.K., Borrelli D.A., Matsuda A., Parasramka M., Shukla N., Lee D.D., Patel T. (2017). Extracellular vesicles from bone marrow-derived mesenchymal stem cells protect against murine hepatic ischemia/reperfusion injury. Liver Transpl..

[26] Tamura R., Uemoto S., Tabata Y. (2016). Immunosuppressive effect of mesenchymal stem cell-derived exosomes on a concanavalin A-induced liver injury model. Inflamm. Regen..

[27] Rigo F., De Stefano N., Navarro-Tableros V., David E., Rizza G., Catalano G., Gilbo N., Maione F., Gonella F., Roggio D. (2018). Extracellular Vesicles from Human Liver Stem Cells Reduce Injury in an Ex Vivo Normothermic Hypoxic Rat Liver Perfusion Model. Transplantation.

[28] Li T., Yan Y., Wang B., Qian H., Zhang X., Shen L., Wang M., Zhou Y., Zhu W., Li W. (2013). Exosomes derived from human umbilical cord mesenchymal stem cells alleviate liver fibrosis. Stem Cells Dev..

[29] Qu Y., Zhang Q., Cai X., Li F., Ma Z., Xu M., Lu L. (2017). Exosomes derived from miR-181-5p-modified adipose-derived mesenchymal stem cells prevent liver fibrosis via autophagy activation. J. Cell. Mol. Med..

[30] Yan Y., Jiang W., Tan Y., Zou S., Zhang H., Mao F., Gong A., Qian H., Xu W. (2017). hucMSC Exosome-Derived GPX1 Is Required for the Recovery of Hepatic Oxidant Injury. Mol. Ther..

[31] Haga H., Yan I.K., Takahashi K., Matsuda A., Patel T. (2017). Extracellular Vesicles from Bone Marrow-Derived Mesenchymal Stem Cells Improve Survival from Lethal Hepatic Failure in Mice. Stem Cells Transl. Med..

[32] Sun Y., Shi H., Yin S., Ji C., Zhang X., Zhang B., Wu P., Shi Y., Mao F., Yan Y. (2018). Human Mesenchymal Stem Cell Derived Exosomes Alleviate Type 2 Diabetes Mellitus by Reversing Peripheral Insulin Resistance and Relieving beta-Cell Destruction. ACS Nano.

[33] Nie W., Ma X., Yang C., Chen Z., Rong P., Wu M., Jiang J., Tan M., Yi S., Wang W. (2018). Human mesenchymal-stem-cells-derived exosomes are important in enhancing porcine islet resistance to hypoxia. Xenotransplantation.

[34] Shigemoto-Kuroda T., Oh J.Y., Kim D.K., Jeong H.J., Park S.Y., Lee H.J., Park J.W., Kim T.W., An S.Y., Prockop D.J. (2017). MSC-derived Extracellular Vesicles Attenuate Immune Responses in Two Autoimmune Murine Models: Type 1 Diabetes and Uveoretinitis. Stem Cell Rep..

[35] Favaro E., Carpanetto A., Lamorte S., Fusco A., Caorsi C., Deregibus M.C., Bruno S., Amoroso A., Giovarelli M., Porta M. (2014). Human mesenchymal stem cell-derived microvesicles modulate T cell response to islet antigen glutamic acid decarboxylase in patients with type 1 diabetes. Diabetologia.

[36] Jiang Z.Z., Liu Y.M., Niu X., Yin J.Y., Hu B., Guo S.C., Fan Y., Wang Y., Wang N.S. (2016). Exosomes secreted by human urine-derived stem cells could prevent kidney complications from type I diabetes in rats. Stem Cell. Res. Ther..

[37] Nakano M., Nagaishi K., Konari N., Saito Y., Chikenji T., Mizue Y., Fujimiya M. (2016). Bone marrow-derived mesenchymal stem cells improve diabetes-induced cognitive impairment by exosome transfer into damaged neurons and astrocytes. Sci. Rep..

[38] Zhang W., Wang Y., Kong Y. (2019). Exosomes Derived from Mesenchymal Stem Cells Modulate miR-126 to Ameliorate Hyperglycemia-Induced Retinal Inflammation Via Targeting HMGB1. Investig. Ophthalmol. Vis. Sci..

[39] He G.H., Zhang W., Ma Y.X., Yang J., Chen L., Song J., Chen S. (2018). Mesenchymal stem cells-derived exosomes ameliorate blue light stimulation in retinal pigment epithelium cells and retinal laser injury by VEGF-dependent mechanism. Int. J. Ophthalmol..

[40] Moisseiev E., Anderson J.D., Oltjen S., Goswami M., Zawadzki R.J., Nolta J.A., Park S.S. (2017). Protective Effect of Intravitreal Administration of Exosomes Derived from Mesenchymal Stem Cells on Retinal Ischemia. Curr. Eye Res..

[41] Shen T., Zheng Q.Q., Shen J., Li Q.S., Song X.H., Luo H.B., Hong C.Y., Yao K. (2018). Effects of Adipose-derived Mesenchymal Stem Cell Exosomes on Corneal Stromal Fibroblast Viability and Extracellular Matrix Synthesis. Chin. Med. J. (Engl.).

[42] Samaeekia R., Rabiee B., Putra I., Shen X., Park Y.J., Hematti P., Eslani M., Djalilian A.R. (2018). Effect of Human Corneal Mesenchymal Stromal Cell-derived Exosomes on Corneal Epithelial Wound Healing. Investig. Ophthalmol. Vis. Sci..

[43] Yuan X., Li D., Chen X., Han C., Xu L., Huang T., Dong Z., Zhang M. (2017). Extracellular vesicles from human-induced pluripotent stem cell-derived mesenchymal stromal cells (hiPSC-MSCs) protect against renal ischemia/reperfusion injury via delivering specificity protein (SP1) and transcriptional activating of sphingosine kinase 1 and inhibiting necroptosis. Cell Death Dis..

[44] Ranghino A., Bruno S., Bussolati B., Moggio A., Dimuccio V., Tapparo M., Biancone L., Gontero P., Frea B., Camussi G. (2017). The effects of glomerular and tubular renal progenitors and derived extracellular vesicles on recovery from acute kidney injury. Stem Cell. Res. Ther..

[45] Jia H., Liu W., Zhang B., Wang J., Wu P., Tandra N., Liang Z., Ji C., Yin L., Hu X. (2018). HucMSC exosomes-delivered 14-3-3zeta enhanced autophagy via modulation of ATG16L in preventing cisplatin-induced acute kidney injury. Am. J. Transl. Res..

[46] Tomasoni S., Longaretti L., Rota C., Morigi M., Conti S., Gotti E., Capelli C., Introna M., Remuzzi G., Benigni A. (2013). Transfer of growth factor receptor mRNA via exosomes unravels the regenerative effect of mesenchymal stem cells. Stem Cells Dev..

[47] Eirin A., Zhu X.Y., Puranik A.S., Tang H., McGurren K.A., van Wijnen A.J., Lerman A., Lerman L.O. (2017). Mesenchymal stem cell-derived extracellular vesicles attenuate kidney inflammation. Kidney Int..

[48] Wang B., Yao K., Huuskes B.M., Shen H.H., Zhuang J., Godson C., Brennan E.P., Wilkinson-Berka J.L., Wise A.F., Ricardo S.D. (2016). Mesenchymal Stem Cells Deliver Exogenous MicroRNA-let7c via Exosomes to Attenuate Renal Fibrosis. Mol. Ther..

[49] Kim Y.J., Yoo S.M., Park H.H., Lim H.J., Kim Y.L., Lee S., Seo K.W., Kang K.S. (2017). Exosomes derived from human umbilical cord blood mesenchymal stem cells stimulates rejuvenation of human skin. Biochem. Biophys. Res. Commun..

[50] Zhang B., Wang M., Gong A., Zhang X., Wu X., Zhu Y., Shi H., Wu L., Zhu W., Qian H. (2015). HucMSC-Exosome Mediated-Wnt4 Signaling Is Required for Cutaneous Wound Healing. Stem Cells.

[51] Shi H., Xu X., Zhang B., Xu J., Pan Z., Gong A., Zhang X., Li R., Sun Y., Yan Y. (2017). 3,3’-Diindolylmethane stimulates exosomal Wnt11 autocrine signaling in human umbilical cord mesenchymal stem cells to enhance wound healing. Theranostics.

[52] Fang S., Xu C., Zhang Y., Xue C., Yang C., Bi H., Qian X., Wu M., Ji K., Zhao Y. (2016). Umbilical Cord-Derived Mesenchymal Stem Cell-Derived Exosomal MicroRNAs Suppress Myofibroblast Differentiation by Inhibiting the Transforming Growth Factor-beta/SMAD2 Pathway During Wound Healing. Stem Cells Transl. Med..

[53] Chen C.Y., Rao S.S., Ren L., Hu X.K., Tan Y.J., Hu Y., Luo J., Liu Y.W., Yin H., Huang J. (2018). Exosomal DMBT1 from human urine-derived stem cells facilitates diabetic wound repair by promoting angiogenesis. Theranostics.

[54] Oh M., Lee J., Kim Y.J., Rhee W.J., Park J.H. (2018). Exosomes Derived from Human Induced Pluripotent Stem Cells Ameliorate the Aging of Skin Fibroblasts. Int. J. Mol. Sci..

[55] Cho B.S., Kim J.O., Ha D.H., Yi Y.W. (2018). Exosomes derived from human adipose tissue-derived mesenchymal stem cells alleviate atopic dermatitis. Stem Cell. Res. Ther..

[56] Qi X., Zhang J., Yuan H., Xu Z., Li Q., Niu X., Hu B., Wang Y., Li X. (2016). Exosomes Secreted by Human-Induced Pluripotent Stem Cell-Derived Mesenchymal Stem Cells Repair Critical-Sized Bone Defects through Enhanced Angiogenesis and Osteogenesis in Osteoporotic Rats. Int. J. Biol. Sci..

[57] Liu X., Li Q., Niu X., Hu B., Chen S., Song W., Ding J., Zhang C., Wang Y. (2017). Exosomes Secreted from Human-Induced Pluripotent Stem Cell-Derived Mesenchymal Stem Cells Prevent Osteonecrosis of the Femoral Head by Promoting Angiogenesis. Int. J. Biol. Sci..

[58] Zhang Y., Hao Z., Wang P., Xia Y., Wu J., Xia D., Fang S., Xu S. (2019). Exosomes from human umbilical cord mesenchymal stem cells enhance fracture healing through HIF-1alpha-mediated promotion of angiogenesis in a rat model of stabilized fracture. Cell Prolif..

[59] Kim M., Steinberg D.R., Burdick J.A., Mauck R.L. (2019). Extracellular vesicles mediate improved functional outcomes in engineered cartilage produced from MSC/chondrocyte cocultures. Proc. Natl. Acad. Sci. USA.

[60] Vonk L.A., van Dooremalen S.F.J., Liv N., Klumperman J., Coffer P.J., Saris D.B.F., Lorenowicz M.J. (2018). Mesenchymal Stromal/stem Cell-derived Extracellular Vesicles Promote Human Cartilage Regeneration In Vitro. Theranostics.

[61] Otsuru S., Desbourdes L., Guess A.J., Hofmann T.J., Relation T., Kaito T., Dominici M., Iwamoto M., Horwitz E.M. (2018). Extracellular vesicles released from mesenchymal stromal cells stimulate bone growth in osteogenesis imperfecta. Cytotherapy.

[62] Zhang S., Chuah S.J., Lai R.C., Hui J.H.P., Lim S.K., Toh W.S. (2018). MSC exosomes mediate cartilage repair by enhancing proliferation, attenuating apoptosis and modulating immune reactivity. Biomaterials.

[63] Wang Y., Yu D., Liu Z., Zhou F., Dai J., Wu B., Zhou J., Heng B.C., Zou X.H., Ouyang H. (2017). Exosomes from embryonic mesenchymal stem cells alleviate osteoarthritis through balancing synthesis and degradation of cartilage extracellular matrix. Stem Cell. Res. Ther..

[64] Mao G., Zhang Z., Hu S., Chang Z., Huang Z., Liao W., Kang Y. (2018). Exosomes derived from miR-92a-3p-overexpressing human mesenchymal stem cells enhance chondrogenesis and suppress cartilage degradation via targeting WNT5A. Stem Cell. Res. Ther..

[65] Zhu L.L., Huang X., Yu W., Chen H., Chen Y., Dai Y.T. (2018). Transplantation of adipose tissue-derived stem cell-derived exosomes ameliorates erectile function in diabetic rats. Andrologia.

[66] Huang B., Lu J., Ding C., Zou Q., Wang W., Li H. (2018). Exosomes derived from human adipose mesenchymal stem cells improve ovary function of premature ovarian insufficiency by targeting SMAD. Stem Cell. Res. Ther..

[67] Zhao H., Shang Q., Pan Z., Bai Y., Li Z., Zhang H., Zhang Q., Guo C., Zhang L., Wang Q. (2018). Exosomes From Adipose-Derived Stem Cells Attenuate Adipose Inflammation and Obesity Through Polarizing M2 Macrophages and Beiging in White Adipose Tissue. Diabetes.

[68] Ratajczak J., Miekus K., Kucia M., Zhang J., Reca R., Dvorak P., Ratajczak M.Z. (2006). Embryonic stem cell-derived microvesicles reprogram hematopoietic progenitors: Evidence for horizontal transfer of mRNA and protein delivery. Leukemia.

[69] Gradilla A.C., Gonzalez E., Seijo I., Andres G., Bischoff M., Gonzalez-Mendez L., Sanchez V., Callejo A., Ibanez C., Guerra M. (2014). Exosomes as Hedgehog carriers in cytoneme-mediated transport and secretion. Nat. Commun..

[70] McBride J.D., Rodriguez-Menocal L., Guzman W., Candanedo A., Garcia-Contreras M., Badiavas E.V. (2017). Bone Marrow Mesenchymal Stem Cell-Derived CD63(+) Exosomes Transport Wnt3a Exteriorly and Enhance Dermal Fibroblast Proliferation, Migration, and Angiogenesis In Vitro. Stem Cells Dev..

[71] Mathiyalagan P., Liang Y., Kim D., Misener S., Thorne T., Kamide C.E., Klyachko E., Losordo D.W., Hajjar R.J., Sahoo S. (2017). Angiogenic Mechanisms of Human CD34(+) Stem Cell Exosomes in the Repair of Ischemic Hindlimb. Circ. Res..

[72] Zhang Y., Chopp M., Meng Y., Katakowski M., Xin H., Mahmood A., Xiong Y. (2015). Effect of exosomes derived from multipluripotent mesenchymal stromal cells on functional recovery and neurovascular plasticity in rats after traumatic brain injury. J. Neurosurg..

[73] Mead B., Tomarev S. (2017). Bone Marrow-Derived Mesenchymal Stem Cells-Derived Exosomes Promote Survival of Retinal Ganglion Cells Through miRNA-Dependent Mechanisms. Stem Cells Transl. Med..

[74] Yao J., Zheng J., Cai J., Zeng K., Zhou C., Zhang J., Li S., Li H., Chen L., He L. (2019). Extracellular vesicles derived from human umbilical cord mesenchymal stem cells alleviate rat hepatic ischemia-reperfusion injury by suppressing oxidative stress and neutrophil inflammatory response. FASEB J..

[75] Sedrakyan S., Villani V., Da Sacco S., Tripuraneni N., Porta S., Achena A., Lavarreda-Pearce M., Petrosyan A., Soloyan H., Filippo R.E. (2017). Amniotic fluid stem cell-derived vesicles protect from VEGF-induced endothelial damage. Sci. Rep..

[76] Fujii S., Miura Y., Fujishiro A., Shindo T., Shimazu Y., Hirai H., Tahara H., Takaori-Kondo A., Ichinohe T., Maekawa T. (2018). Graft-Versus-Host Disease Amelioration by Human Bone Marrow Mesenchymal Stromal/Stem Cell-Derived Extracellular Vesicles Is Associated with Peripheral Preservation of Naive T Cell Populations. Stem Cells.

[77] Li X., Liu L., Yang J., Yu Y., Chai J., Wang L., Ma L., Yin H. (2016). Exosome Derived From Human Umbilical Cord Mesenchymal Stem Cell Mediates MiR-181c Attenuating Burn-induced Excessive Inflammation. EBioMedicine.

[78] Song Y., Dou H., Li X., Zhao X., Li Y., Liu D., Ji J., Liu F., Ding L., Ni Y. (2017). Exosomal miR-146a Contributes to the Enhanced Therapeutic Efficacy of Interleukin-1beta-Primed Mesenchymal Stem Cells Against Sepsis. Stem Cells.

[79] Fan Y., Herr F., Vernochet A., Mennesson B., Oberlin E., Durrbach A. (2019). Human Fetal Liver Mesenchymal Stem Cell-Derived Exosomes Impair Natural Killer Cell Function. Stem Cells Dev..

[80] Lee M.W., Ryu S., Kim D.S., Lee J.W., Sung K.W., Koo H.H., Yoo K.H. (2019). Mesenchymal stem cells in suppression or progression of hematologic malignancy: Current status and challenges. Leukemia.

[81] Christodoulou I., Goulielmaki M., Devetzi M., Panagiotidis M., Koliakos G., Zoumpourlis V. (2018). Mesenchymal stem cells in preclinical cancer cytotherapy: A systematic review. Stem Cell. Res. Ther..

[82] Hendijani F., Javanmard S.H., Sadeghi-aliabadi H. (2015). Human Wharton’s jelly mesenchymal stem cell secretome display antiproliferative effect on leukemia cell line and produce additive cytotoxic effect in combination with doxorubicin. Tissue Cell.

[83] Ho J.H., Chen Y.F., Ma W.H., Tseng T.C., Chen M.H., Lee O.K. (2011). Cell contact accelerates replicative senescence of human mesenchymal stem cells independent of telomere shortening and p53 activation: Roles of Ras and oxidative stress. Cell Transplant..

[84] Colter D.C., Class R., DiGirolamo C.M., Prockop D.J. (2000). Rapid expansion of recycling stem cells in cultures of plastic-adherent cells from human bone marrow. Proc. Natl. Acad. Sci. USA.

[85] Patel D.B., Gray K.M., Santharam Y., Lamichhane T.N., Stroka K.M., Jay S.M. (2017). Impact of cell culture parameters on production and vascularization bioactivity of mesenchymal stem cell-derived extracellular vesicles. Bioeng. Transl. Med..

[86] Kulkarni R., Bajaj M., Ghode S., Jalnapurkar S., Limaye L., Kale V.P. (2018). Intercellular Transfer of Microvesicles from Young Mesenchymal Stromal Cells Rejuvenates Aged Murine Hematopoietic Stem Cells. Stem Cells.

[87] Wang X., Omar O., Vazirisani F., Thomsen P., Ekstrom K. (2018). Mesenchymal stem cell-derived exosomes have altered microRNA profiles and induce osteogenic differentiation depending on the stage of differentiation. PLoS ONE.

[88] Ruan X.F., Ju C.W., Shen Y., Liu Y.T., Kim I.M., Yu H., Weintraub N., Wang X.L., Tang Y. (2018). Suxiao Jiuxin pill promotes exosome secretion from mouse cardiac mesenchymal stem cells in vitro. Acta Pharmacol. Sin..

[89] Harting M.T., Srivastava A.K., Zhaorigetu S., Bair H., Prabhakara K.S., Toledano Furman N.E., Vykoukal J.V., Ruppert K.A., Cox C.S., Olson S.D. (2018). Inflammation-Stimulated Mesenchymal Stromal Cell-Derived Extracellular Vesicles Attenuate Inflammation. Stem Cells.

[90] Patel D.B., Santoro M., Born L.J., Fisher J.P., Jay S.M. (2018). Towards rationally designed biomanufacturing of therapeutic extracellular vesicles: Impact of the bioproduction microenvironment. Biotechnol. Adv..

[91] Watson D.C., Bayik D., Srivatsan A., Bergamaschi C., Valentin A., Niu G., Bear J., Monninger M., Sun M., Morales-Kastresana A. (2016). Efficient production and enhanced tumor delivery of engineered extracellular vesicles. Biomaterials.

[92] Jarmalaviciute A., Tunaitis V., Pivoraite U., Venalis A., Pivoriunas A. (2015). Exosomes from dental pulp stem cells rescue human dopaminergic neurons from 6-hydroxy-dopamine-induced apoptosis. Cytotherapy.

[93] Zhang Y., Chopp M., Zhang Z.G., Katakowski M., Xin H., Qu C., Ali M., Mahmood A., Xiong Y. (2017). Systemic administration of cell-free exosomes generated by human bone marrow derived mesenchymal stem cells cultured under 2D and 3D conditions improves functional recovery in rats after traumatic brain injury. Neurochem. Int..

[94] Abello J., Nguyen T.D.T., Marasini R., Aryal S., Weiss M.L. (2019). Biodistribution of gadolinium- and near infrared-labeled human umbilical cord mesenchymal stromal cell-derived exosomes in tumor bearing mice. Theranostics.

[95] Wiklander O.P., Nordin J.Z., O’Loughlin A., Gustafsson Y., Corso G., Mager I., Vader P., Lee Y., Sork H., Seow Y. (2015). Extracellular vesicle in vivo biodistribution is determined by cell source, route of administration and targeting. J. Extracell Vesicles.

[96] Valadi H., Ekstrom K., Bossios A., Sjostrand M., Lee J.J., Lotvall J.O. (2007). Exosome-mediated transfer of mRNAs and microRNAs is a novel mechanism of genetic exchange between cells. Nat. Cell Biol..

[97] Xin H., Wang F., Li Y., Lu Q.E., Cheung W.L., Zhang Y., Zhang Z.G., Chopp M. (2017). Secondary Release of Exosomes From Astrocytes Contributes to the Increase in Neural Plasticity and Improvement of Functional Recovery After Stroke in Rats Treated With Exosomes Harvested From MicroRNA 133b-Overexpressing Multipotent Mesenchymal Stromal Cells. Cell Transpl..

[98] Ju Z., Ma J., Wang C., Yu J., Qiao Y., Hei F. (2017). Exosomes from iPSCs Delivering siRNA Attenuate Intracellular Adhesion Molecule-1 Expression and Neutrophils Adhesion in Pulmonary Microvascular Endothelial Cells. Inflammation.

[99] Kojima R., Bojar D., Rizzi G., Hamri G.C., El-Baba M.D., Saxena P., Auslander S., Tan K.R., Fussenegger M. (2018). Designer exosomes produced by implanted cells intracerebrally deliver therapeutic cargo for Parkinson’s disease treatment. Nat. Commun..

[100] Chao M.P., Weissman I.L., Majeti R. (2012). The CD47-SIRPalpha pathway in cancer immune evasion and potential therapeutic implications. Curr. Opin. Immunol..

[101] Cao Y., Wu T., Zhang K., Meng X., Dai W., Wang D., Dong H., Zhang X. (2019). Engineered Exosome-Mediated Near-Infrared-II Region V2C Quantum Dot Delivery for Nucleus-Target Low-Temperature Photothermal Therapy. ACS Nano.

[102] Sun D., Zhuang X., Xiang X., Liu Y., Zhang S., Liu C., Barnes S., Grizzle W., Miller D., Zhang H.G. (2010). A novel nanoparticle drug delivery system: The anti-inflammatory activity of curcumin is enhanced when encapsulated in exosomes. Mol. Ther..

[103] Pascucci L., Cocce V., Bonomi A., Ami D., Ceccarelli P., Ciusani E., Vigano L., Locatelli A., Sisto F., Doglia S.M. (2014). Paclitaxel is incorporated by mesenchymal stromal cells and released in exosomes that inhibit in vitro tumor growth: A new approach for drug delivery. J. Control. Release.

[104] Del Fattore A., Luciano R., Saracino R., Battafarano G., Rizzo C., Pascucci L., Alessandri G., Pessina A., Perrotta A., Fierabracci A. (2015). Differential effects of extracellular vesicles secreted by mesenchymal stem cells from different sources on glioblastoma cells. Expert Opin. Biol. Ther..

[105] Perteghella S., Crivelli B., Catenacci L., Sorrenti M., Bruni G., Necchi V., Vigani B., Sorlini M., Torre M.L., Chlapanidas T. (2017). Stem cell-extracellular vesicles as drug delivery systems: New frontiers for silk/curcumin nanoparticles. Int. J. Pharm..

[106] Tian Y., Li S., Song J., Ji T., Zhu M., Anderson G.J., Wei J., Nie G. (2014). A doxorubicin delivery platform using engineered natural membrane vesicle exosomes for targeted tumor therapy. Biomaterials.

[107] Ran L., Tan X., Li Y., Zhang H., Ma R., Ji T., Dong W., Tong T., Liu Y., Chen D. (2016). Delivery of oncolytic adenovirus into the nucleus of tumorigenic cells by tumor microparticles for virotherapy. Biomaterials.

[108] Lawler S.E., Speranza M.C., Cho C.F., Chiocca E.A. (2017). Oncolytic Viruses in Cancer Treatment: A Review. JAMA Oncol..

[109] Harrington K., Freeman D.J., Kelly B., Harper J., Soria J.C. (2019). Optimizing oncolytic virotherapy in cancer treatment. Nat. Rev. Drug Discov..

[110] Garofalo M., Villa A., Crescenti D., Marzagalli M., Kuryk L., Limonta P., Mazzaferro V., Ciana P. (2019). Heterologous and cross-species tropism of cancer-derived extracellular vesicles. Theranostics.

[111] Garofalo M., Villa A., Rizzi N., Kuryk L., Mazzaferro V., Ciana P. (2018). Systemic Administration and Targeted Delivery of Immunogenic Oncolytic Adenovirus Encapsulated in Extracellular Vesicles for Cancer Therapies. Viruses.

[112] Garofalo M., Villa A., Rizzi N., Kuryk L., Rinner B., Cerullo V., Yliperttula M., Mazzaferro V., Ciana P. (2019). Extracellular vesicles enhance the targeted delivery of immunogenic oncolytic adenovirus and paclitaxel in immunocompetent mice. J. Control. Release.

[113] Sonabend A.M., Ulasov I.V., Tyler M.A., Rivera A.A., Mathis J.M., Lesniak M.S. (2008). Mesenchymal stem cells effectively deliver an oncolytic adenovirus to intracranial glioma. Stem Cells.

[114] Hoyos V., Del Bufalo F., Yagyu S., Ando M., Dotti G., Suzuki M., Bouchier-Hayes L., Alemany R., Brenner M.K. (2015). Mesenchymal Stromal Cells for Linked Delivery of Oncolytic and Apoptotic Adenoviruses to Non-small-cell Lung Cancers. Mol. Ther..

